# Nucleolytic processing of aberrant replication intermediates by an Exo1-Dna2-Sae2 axis counteracts fork collapse-driven chromosome instability

**DOI:** 10.1093/nar/gkw858

**Published:** 2016-09-26

**Authors:** Arianna Colosio, Camilla Frattini, Grazia Pellicanò, Sara Villa-Hernández, Rodrigo Bermejo

**Affiliations:** 1 The F.I.R.C. Institute of Molecular Oncology (IFOM) Foundation, Via Adamello 16, 20139 Milan, Italy; 2 Instituto de Biología Funcional y Genómica (IBFG-CSIC), Universidad de Salamanca, Calle Zacarías González 2, 37007 Salamanca, Spain; 3 Centro de Investigaciones Biológicas (CIB-CSIC), Calle Ramiro de Maeztu 9, 28040 Madrid, Spain

## Abstract

Problems during DNA replication underlie genomic instability and drive malignant transformation. The DNA damage checkpoint stabilizes stalled replication forks thus counteracting aberrant fork transitions, DNA breaks and chromosomal rearrangements. We analyzed fork processing in checkpoint deficient cells by coupling psoralen crosslinking with replication intermediate two-dimensional gel analysis. This revealed a novel role for Exo1 nuclease in resecting reversed replication fork structures and counteracting the accumulation of aberrant intermediates resembling fork cleavage products. Genetic analyses demonstrated a functional interplay of Exo1 with Mus81, Dna2 and Sae2 nucleases in promoting cell survival following replication stress, suggestive of concerted nucleolytic processing of stalled forks. While Mus81 and other Structure Specific Endonucleases do not contribute to obvious collapsed fork transitions, Dna2 promotes reversed fork resection likely by facilitating Exo1 access to nascent strands. Instead, Sae2 cooperates with Exo1 in counteracting putative fork cleavage events linked to double strand breaks formation and increased gross chromosomal rearrangement rates. Our data indicate that in checkpoint deficient cells diverse nuclease activities interface to eliminate aberrant replication intermediates and prevent chromosome instability.

## INTRODUCTION

Preventing errors during DNA replication is essential to guarantee the correct transmission of genetic information. However, chromosome replication is frequently challenged by natural and exogenous impediments. Conditions that perturb DNA synthesis or replication fork progression, generally defined as ‘replication stress’, favor genomic instability, a hallmark of cancer cells (1–5). The DNA damage checkpoint is a key mechanism that eukaryotic cells have evolved to protect genome integrity during replication (6,7). The checkpoint response is coordinated by the central protein kinases Mec1/ATR and Rad53/CHK2, which ensure the stabilization and timely restart of stalled forks, essential in turn for viability and genome integrity maintenance in cells experiencing replication stress (8,9).

Replication stress generally results in the uncoupling between DNA synthesis at leading and lagging strands and DNA unwinding by replicative helicases (10). Stalled forks accumulate Replication Protein A (RPA)-covered single stranded DNA (ssDNA) that recruits Mec1/ATR, subsequently triggering Rad53/CHK2 activation (11,12). In checkpoint mutants, stalled replication forks undergo structural transitions that prime chromosomal rearrangements through mechanisms still poorly understood (2,13,14). Rad53 plays a key role in preserving both the structural integrity of replication intermediates and the proficiency for DNA synthesis of stalled forks (6,15–18). In addition, the DNA damage checkpoint preserves genome integrity by modulating chromosome architecture to relieve topological stress (19) and by inhibiting late-origin firing (20).

Recently, replication stress has emerged as a driving force of malignant transformation (4,21). Oncogene activation causes replication fork stalling that leads to over-replication, fork collapse and DNA breaks formation (22–24). In pre-cancerous lesions, the DNA damage response is thought to act as a barrier to tumor progression by driving cells with unstable genomes out of proliferating pools into apoptosis or senescence (25). Thus, mutations inactivating checkpoint factors may promote cancer onset by both enhancing DNA damage resulting from oncogene-induced fork stalling and allowing premalignant cells to evade proliferative restraints (1,21).

In this work we investigated nuclease-mediated stalled fork transitions and their contribution to chromosome instability in checkpoint-deficient budding yeast cells. We found that the Exo1, Dna2 and Sae2 nucleases interplay in processing replication forks and in preventing the accumulation of aberrantly shaped intermediates likely resulting from branch cleavage events. The abnormal fork transitions counteracted by Exo1/Dna2 and Sae2 are linked to DNA breaks formation and increased rates of chromosomal rearrangements. Our findings indicate that upon fork collapse owing to checkpoint deficiencies a network of nucleases clears aberrant intermediates to prevent transitions leading to chromosome breakage and instability.

## MATERIALS AND METHODS

### *Saccharomyces cerevisiae* strains

All strains are derivative from *W303* and are listed in Supplementary Table S1. Gene deletions were obtained through the one-step polymerase chain reaction method (26).

### Growing conditions, synchronization and western blotting

Yeast strains were grown in YPDA media at 28°C unless otherwise stated. For G1 arrests, early log phase cells were arrested with 4 μg/ml α-Factor and then released in S phase by centrifugation, washing with 1 volume of YP and resuspension in fresh YPDA media containing 200 mM hydroxyurea (HU). For western blot analysis extracts were prepared by Trichloroacetic acid (TCA) precipitation and processed as described (27).

### Two-dimensional gel analysis of replication intermediates

Two-dimensional (2D) gel analysis and *in vivo* psoralen-crosslinking were carried out as described (28–30). Psoralen crosslinking was carried out as follows. In brief, cells were washed, resuspended in sterile water and placed in 6-well multi-well plates on ice and 300 μl of a 0.2 mg/ml 4,5′,8 trimethylpsoralen (Sigma-Aldrich, T-6137) stock solution were added. After incubation for five minutes in the dark, cells were irradiated for 10 min with 366 nm UV light on a Stratalinker (UVP CL-1000; Ultraviolet Crosslinker, LabGear, USA). The crosslinking procedure was repeated four times. Cells were disrupted through mechanical breakage by resuspension in nuclei isolation buffer and vortexing with glass beads. DNA extraction was performed according to the ‘QIAGEN genomic DNA Handbook,’ using genomic-tip 100/G columns. DNA was digested with NcoI restriction enzyme, unless differently stated. Images were acquired using a Phosphoimager Typhoon Trio Instrument (GE Healthcare). Quantification of replication intermediates was performed using Image Quant TL 8.1 software (GE Healthcare). In brief, signal intensities of regions containing the different molecules (i.e. X-spike, cone and Y-spike intermediates) were quantified and the background intensity normalized to the area of each intermediate region was subtracted. Intermediate signals were normalized by the monomer spot signal values for each panel. Bar charts showing quantification data correspond to the representative 2D experiment shown in each figure panel.

### Pulse-field gel electrophoresis

DNA plugs were prepared as described (31). Yeast chromosomes were digested with EagI restriction enzyme and separated by pulse field gel electrophoresis (PFGE) (Chef Mapper, Biorad). Electrophoresis was performed for 9 h at 6 V/cm with 90 s pulses, followed by 9 h with 60 s pulses, in TBE 0.5× at 10°C prior to Southern blotting.

### Gross chromosomal rearrangement (GCR) assay

Fluctuation analysis of 5-FOA and canavanine-resistant cells was used to determine gross chromosomal rearrangement (GCR) rates. Single colonies were used to inoculate seven independent cultures at a concentration of 2.5 × 10^5^ cells/ml in 20–100 ml of YPDA medium and grown at 30°C. When cultures reached saturation, cells were spread on 5-FOA/canavanine-containing plates and, following 1/10 000 dilution, on YPD plates. A maximum of 5 × 10^8^ cells was spread on 140 mm plates and incubated at 30°C. After 14 days the number of 5-FOA/Can-resistant colonies (r) was counted, as well as the total number of viable cells (Nt) derived from the number of colonies grown on YPD. The GCR rate (M) as well as the upper and lower 95% confidence intervals (95% CI) were calculated from r and Nt with FALCOR (32) using the Ma-Sadri-Sarkar Maximum Likely Estimator (MSS-MLE) method.

## RESULTS

### Exo1 resects reversed nascent strands and counteracts aberrant transitions at collapsed forks

HU induces replication stress by depleting dNTP pools and inhibiting replicative polymerases. In cells exposed to 200 mM HU, replication forks progress slowly for few kilobases before stalling (15,31). Electron microscopy (EM) analysis showed that in *rad53-K227A* checkpoint defective mutants stalled forks accumulate unusual replication intermediates characterized by nascent strand annealing (fork reversal) or extended ssDNA stretches (10). Such collapsed forks fail to further synthesize DNA and are engaged in transitions priming chromosomal rearrangements, through yet poorly understood mechanisms (16).

A key factor determining the fate of collapsed forks is the Exo1 5′-3′ exonuclease belonging to the Rad2 family (33–35). In *rad53* mutants, Exo1 ablation enhances fork reversal and counteracts the accumulation of ssDNA gap-containing intermediates. These phenotypes are related to direct exonucleolytic processing of nascent strands by Exo1, which generates intermediates containing extensive ssDNA thought to preclude strand annealing during fork reversal (33). Noteworthy, inactivation of Exo1 does not to fully suppress aberrant intermediates accumulation in checkpoint deficient cells (33), suggesting the involvement of additional factors in fork processing. Identifying these factors is essential for understanding the mechanisms leading to chromosomal instability in cells with an impaired checkpoint function.

We investigated collapsed fork transitions using neutral-neutral two-dimensional (2D) gel electrophoresis, which have proven a powerful tool for studying replication intermediates forming at collapsed forks (15,30,36). Of note, previous 2D gel analysis failed to evidence major abnormalities in the replication intermediates of HU-treated *rad53 exo1Δ* mutants (33). This apparent inconsistency with EM data may be explained by branch migration events occurring during genomic DNA extraction for 2D gels, which would convert aberrantly shaped structures into simpler shaped molecules. We therefore coupled 2D gel analysis with psoralen crosslinking to preserve the *in vivo* structure of stalled fork intermediates.

**Figure 1.**
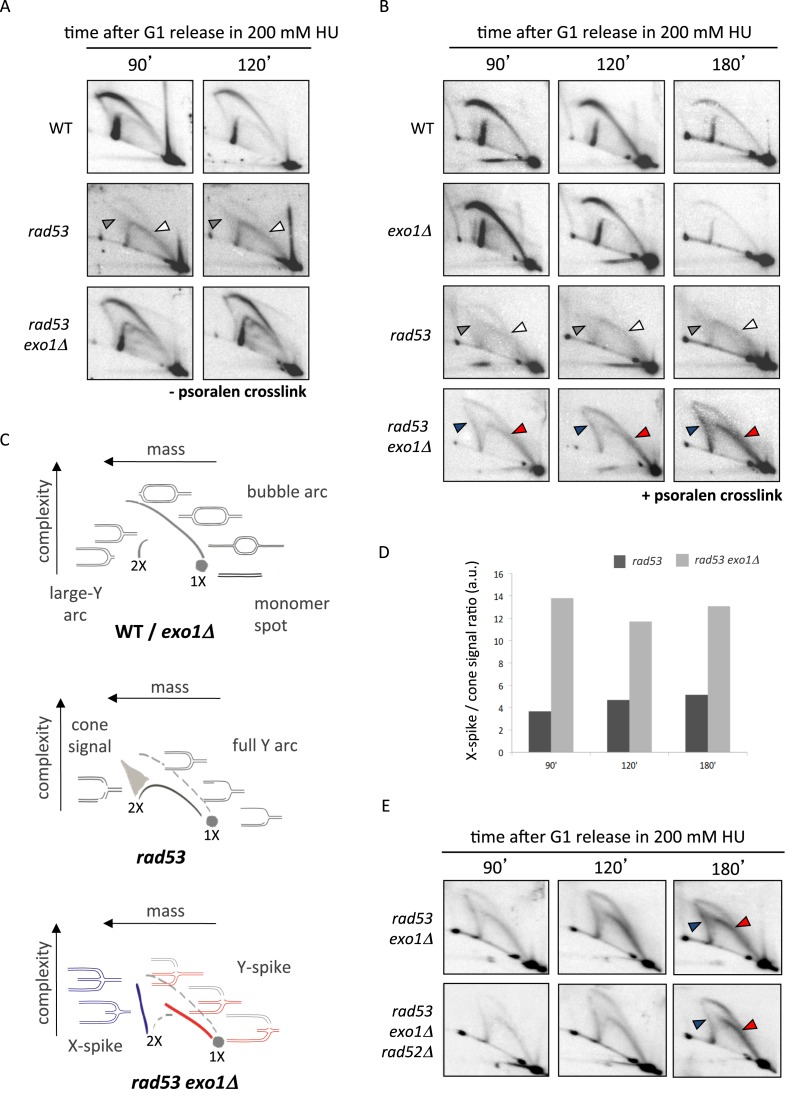
Psoralen two-dimensional (2D) gel analysis of collapsed forks transitions in checkpoint and Exo1 deficient cells. 2D gel analysis of untreated (**A**) and psoralen-crosslinked (**B**). Replication intermediates from wild-type (WT), *exo1Δ, rad53-K227A* (*rad53*) and *rad53-K227A exo1Δ* (*rad53 exo1Δ*) cells collected at the indicated times after release from G1 into S-phase in 200 mM HU. Gray and white arrowheads indicate, respectively, cone and full Y arc signals in *rad53* cells. Arrowheads indicate X-spike and Y-spike signals in *rad53 exo1Δ* cells. (**C**) Schematic interpretation of the 2D gel patterns observed in WT/*exo1Δ, rad53* and *rad53exo1Δ* HU treated cells. (**D**) Histogram plot of the ratios of X-spike/cone signals quantified from 2D gels shown in panel B. (**E**) 2D gel analysis of *rad53 exo1Δ* and *rad53 exo1Δ rad52Δ* mutants at the indicated times after release from G1 into S-phase in 200 mM HU.

We first examined Exo1 contribution to collapsed fork transitions by visualizing intermediates emanating from the *ARS305* early replication origin (37). Wild-type (WT), *exo1Δ, rad53-K227A* (*rad53*) and *rad53-K227A exo1Δ* (*rad53 exo1Δ*) cells were released into a synchronous S phase in the presence of 200 mM HU and 2D gels were performed on genomic DNA extracted from both untreated and psoralen-crosslinked cells (Figure 1A and B). Under either condition, WT cells showed bubble and large Y-shaped intermediates owing to *ARS305* firing (see scheme in Figure 1C), which progressively decreased in intensity as forks moved outside of the analyzed fragment. Similar profiles were observed for *exo1* cells (data not shown and Figure 1B). In agreement with previous reports (15,33), non-crosslinked *rad53* cells accumulated intermediates migrating along a cone shaped area (gray arrowheads) and a full Y arc (white arrowheads), which likely correspond to resected reversed forks and extended ssDNA containing Y-shaped molecules (see scheme on Figure 1C), respectively. We note that cone signals may also contain a proportion of unresected reversed forks in which the branching point has not progressed outside the restriction site defining the fragment end. These intermediates are equivalent to those observed by EM (10) and were also detected upon psoralen crosslinking (Figure 1B).

Remarkable differences were observed when analyzing HU-treated *rad53 exo1Δ* cells, as psoralen treatment stabilized two classes of intermediates that escaped detection in conventional 2D gels. Firstly, a sharp spike of X-shaped intermediates accumulated along time (Figure 1B, blue arrowheads). These intermediates migrated as 4-way branched molecules with the properties of unresected reversed forks: fully replicated (2X) mass and a shape complexity directly proportional to the distance of the branching point to the nearest extremity of the fragment (see Figure 1C, in blue). X-spike intermediates, consistent with reversed forks observed by EM (10), are different from sister chromatid junctions (28) that can also be detected in WT cells but are destabilized upon psoralen treatment (our unpublished observations, compare WT panels on Figure 1A and B). Of note, the vast majority of X-shaped reversed fork signals detected in *rad53* cells shifted from putatively resected (cone) to unresected (X-spike) upon Exo1 ablation (Figure 1D), in agreement with a prominent role of Exo1 in processing nascent strands engaged in fork reversal.

A second class of intermediates stabilized by psoralen-crosslinking in *rad53 exo1* cells distributed along a spike of Y-shaped molecules emanating from the linear monomer spot (Figure 1B, red arrowheads). Y-spike intermediates showed a mass ranging from 1X to ∼1.5X that of linear fragment and a shape complexity directly proportional to mass. Such molecules are suggestive of nucleolytic processing and we speculate they might result from cleavage of parental/newly-synthesized branches within reversed forks (Figure 1C, molecules in red). A similar transition likely owing to endonucleolytic processing has been observed at replication forks approaching double strand DNA breaks (DSBs) (30). Of note, X-shaped and Y-spike molecules also accumulated in *rad53 exo1Δ* cells upon *RAD52* ablation (Figure 1E), suggesting that homologous recombination is dispensable for fork reversal and putative cleavage in checkpoint deficient cells. These data evidence that Exo1-mediated resection counteracts the accumulation of distinct molecules upon fork collapse, including a novel type of intermediate likely resulting from cleavage of reversed forks. We note that the persistence of abnormally shaped intermediates might underlie the inability of *exo1* deletion to restore DNA synthesis and viability in *rad53* mutants following HU-induced replication blocks (33,34).

**Figure 2.**
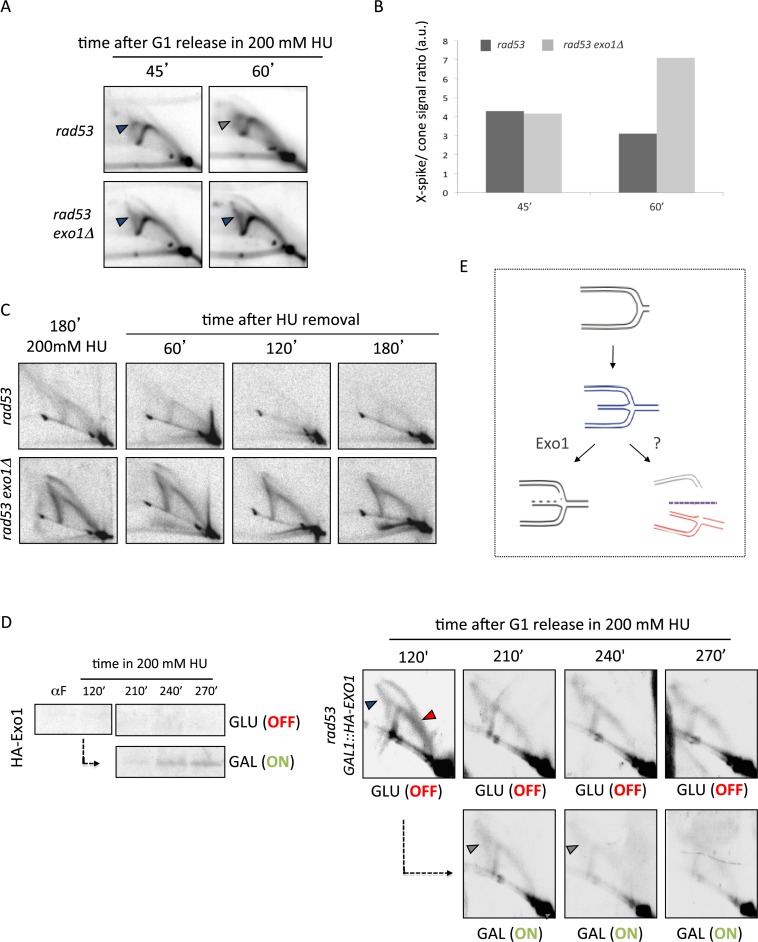
Exo1 resects aberrant intermediates accumulating at collapsed forks in checkpoint mutants. (**A**) 2D gel analysis of *rad53* and *rad53 exo1Δ* cells 45 and 60 min after release from G1 into S-phase in the presence of 200 mM HU. Dark and gray arrowheads indicate X-spike and cone signals, respectively. (**B**) Histogram plot of the ratios of X-spike/Cone signals quantified from 2D gels shown in panel A. (**C**) 2D gel analysis of *rad53* and *rad53 exo1Δ* cells collected at the indicated times after release from a HU-induced replication block. (**D**) *rad53 GAL1::HA-EXO1* cells were released from G1 into S-phase in 200 mM HU and glucose (GLU) to repress Exo1 expression. After 120′ the culture was split and cells were shifted to fresh medium containing either GLU or galactose (GAL) and 200 mM HU. Samples were collected for Western blot detection of Exo1 expression and 2D gel analysis at the indicated time points (**E**) Schematic representation of the impact of Exo1-mediated resection of nascent strands on reversed fork transitions.

We investigated early transitions occurring during fork stalling and collapse. Forty-five minutes after release into S-phase in the presence of HU *rad53* and *rad53 exo1Δ* mutants exhibited canonical bubble and big Y shaped intermediates owing to *ARS305* firing (Figure 2A). Of note, sharp X-spike signals likely owing to unresected reversed forks could be detected in both *rad53* and *rad53 exo1Δ* cells at this stage (blue arrowheads). However, by 60 min X-spike molecules were replaced by a diffuse cone signal in *rad53* cells while further accumulated in Exo1 ablated cells (gray arrowhead), resulting in a marked increase of X-spike to cone signal ratios (Figure 2B). These data revealed that during fork collapse nascent strands annealing can precede their resection by Exo1.

We then analyzed the fate of collapsed forks upon release from a HU-induced replication block (Figure 2C). Aberrant molecule levels gradually decreased in *rad53* cells upon removal of the drug, with cone and full Y arc signals barely appreciable after 120 min. In contrast, *rad53 exo1Δ* cells showed comparable 2D gel patterns along time, showing only a marginal decrease in overall intermediate levels. We then constructed *rad53* cells in which the expression of *EXO1* is under the control of the *GAL1* promoter. This promoter is repressed when cells are grown in the presence of glucose (GLU) and activated in the presence of galactose (GAL). Checkpoint deficient cells released into HU from a G1 block with repressed *EXO1* expression (GLU) showed 2D gel profiles resembling those of *rad53 exo1* cells (Figure 2D). Upon induction of Exo1 expression by switching to GAL containing medium, 2D gel patterns progressively showed an overall reduction of replication intermediate levels including cruciform molecules. These data indicate that Exo1 bears the main activity responsible for clearing non-canonically shaped intermediates forming at collapsed forks, and that it likely does so by generating less complex ssDNA molecules eluding detection in 2D gels. In light of these results, we propose that resection by Exo1 might counteract reversed forks processing into aberrant Y-shaped intermediates by eliminating double stranded DNA structures serving as substrates for nucleolytic cleavage (Figure 2E).

### A network of interplaying nucleases at stalled replication forks

**Figure 3.**
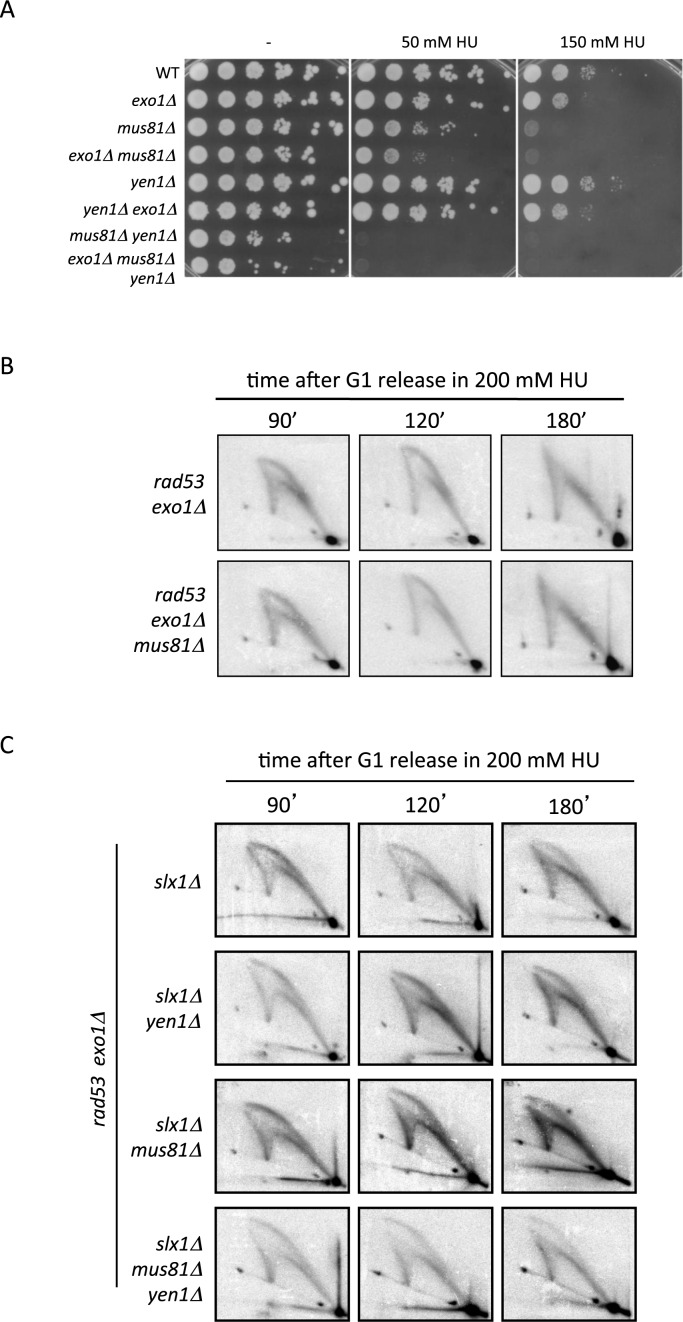
Structure specific endonucleases Mus81, Yen1 and Slx1 are dispensable for the accumulation of aberrant intermediates at collapsed forks. (**A**) Serial dilutions of WT, *exo1Δ, mus81Δ, exo1Δ mus81Δ, yen1Δ, yen1Δ exo1Δ, mus81Δ yen1Δ* and *exo1Δ mus81Δ yen1Δ* cells plated in YPD in the absence (-) or presence of 50 or 150 mM HU. (**B** and **C**) 2D gel analysis of (B) *rad53 exo1Δ* and *rad53 exo1Δ mus81Δ* cells or (C) *rad53 exo1Δ slx1Δ, rad53 exo1Δ slx1Δ yen1Δ, rad53 exo1Δ slx1Δ mus81Δ* and *rad53 slx1Δ exo1Δ mus81Δ yen1Δ* cells at the indicated times after G1 release into S-phase in the presence of 200 mM HU.

In order to reveal additional activities contributing to stalled fork processing, we searched for mutations in nuclease-coding genes conferring synthetic HU sensitivity in combination with *exo1* deletion. We first tested Structure Selective Endonucleases (SSEs) Mus81 and Yen1 (38) that cleave branched structures resembling reversed replication forks (39,40). Cells ablated for Mus81 are sensitive to HU (40,41) (Figure 3A), a phenotype that has been related to a direct role of Mus81 in stalled fork processing (42,43). *mus81* and *yen1* deletions show synthetic sensitivity to replication stress-inducing drugs (40,41,44) (Figure 3A) likely reflecting overlapping roles in the resolution of repair structures arising as a consequence of replication fork stalling (40,45). We found that *exo1Δ* conferred synthetically increased HU sensitivity in combination with *mus81Δ* but not *yen1Δ* (Figure 3A), suggesting that Exo1 interplays with Mus81 in the metabolism of stalled replication forks in a pathway independent of Yen1 function. Ablation of *slx1, rad1* or *saw1* genes, coding for additional factors belonging to SSE complexes able to cleave four-way junctions and fork-like substrates *in vitro* (46–48), did not confer HU sensitivity or alter the sensitivity of *exo1Δ* cells (data not shown).

The synthetic genetic interaction between *exo1* and *mus81* prompted us to analyze whether these nucleases interface in promoting collapsed replication fork transitions. SEEs can act on similar *in vitro* (49) and *in vivo* substrates (45). We therefore extended our 2D gel analysis to *yen1* and *slx1* mutations to cover for possible redundancies in fork processing. We failed to observe differences in the replication intermediates of *rad53* (data not shown) or *rad53 exo1* cells, upon single *mus81* (Figure 3B), *yen1* or double *mus81/yen1* deletion (Supplementary Figure S1). Similarly, *slx1* ablation did not alter the 2D gel profiles of *rad53* (data not shown) or *rad53 exo1* cells, even if combined with *mus81* and/or *yen1* deletions (Figure 3C). We also failed to detect differences in the 2D gel profiles of *rad53* and *rad53 exo1* cells upon ablation of *rad1* or *saw1* (data not shown). These observations indicate that Mus81, Yen1, Slx1 and Rad1 SSE nucleases are dispensable for the aberrant fork transitions observed in checkpoint deficient cells. We note, however, that the synthetic HU sensitivity observed in *exo1 mus81* checkpoint proficient cells points at an overlapping role for these nucleases in stalled fork metabolism, perhaps related to the resolution of fork-derived structures later in the cell cycle (43,50).

**Figure 4.**
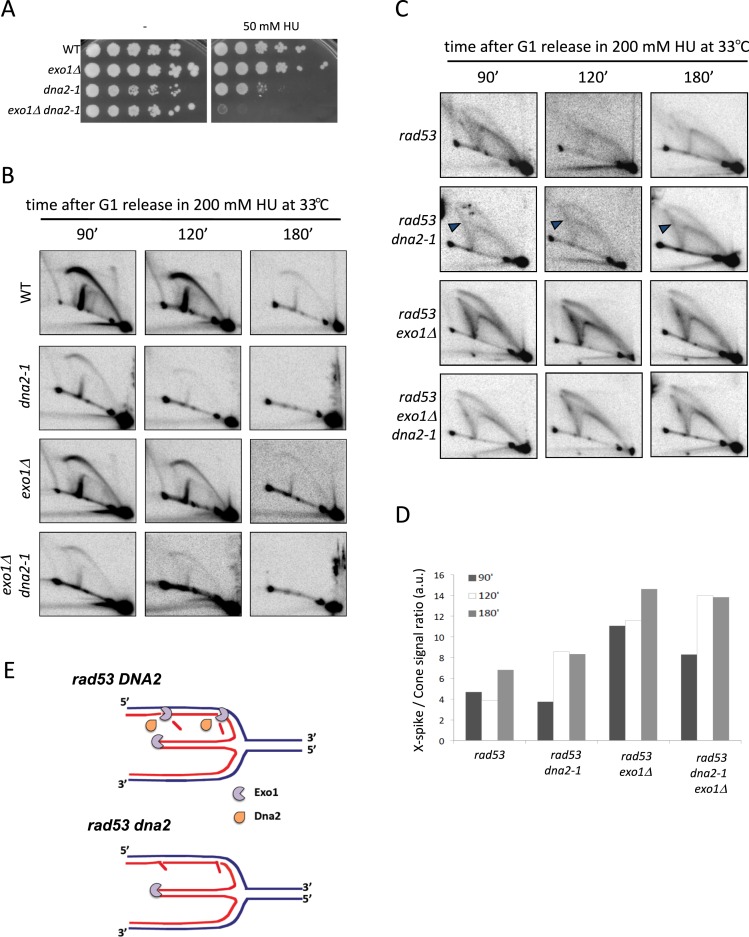
Dna2 influences collapsed fork processing by Exo1. (**A**) Serial dilutions of WT*, exo1Δ, dna2-1* and *dna2-1 exo1Δ* cells plated on YPD in the absence (-) or presence of 50 mM HU and grown at 23°C. (**B**) 2D gel analysis of replication intermediates of WT, *dna2-1, exo1Δ* and *dna2-1 exo1Δ* cells at the indicated times after G1 release into S-phase at 33°C in the presence of 200 mM HU. (**C**) 2D gel analysis of replication intermediates in *rad53, rad53 dna2-1, rad53 exo1Δ* and *rad53 exo1Δ dna2-1* cells at the indicated times after G1 release into S-phase at 33°C in the presence of 200 mM HU. Arrowheads indicate X-spike signals. (**D**) Histogram plot of the ratios of X-spike/Cone signals quantified from 2D gels shown in panel C. (**E**) Schematic representation of nascent strand resection by Exo1 in Dna2 proficient and deficient cells.

We next investigated the contribution of Dna2, a highly conserved nuclease/helicase that plays an essential function in DNA replication by removing long flaps generated by strand displacement during Okazaki fragment synthesis (51–53). We tested the HU sensitivity of cells bearing the *dna2-1* nuclease/helicase defective allele (54) alone or in combination with *exo1* deletion. In agreement with previous reports (55,56), *dna2-1* cells showed a high sensitivity to HU treatment (Figure 4A). We found that *dna2-1* and *exo1Δ* mutations conferred synthetic sensitivity to HU, suggesting that also these nucleases interplay in stalled fork metabolism.

In order to address the contribution of Dna2 to collapsed fork transitions and its interfacing with Exo1, we performed psoralen 2D gels in wild type, *dna2-1, exo1Δ* and *dna2-1 exo1Δ* cells after release into S-phase in the presence of HU (Figure 4B). Experiments were carried out at a semi-permissive temperature for the *dna2-1* allele (data not shown). WT and *exo1Δ* cells fired *ARS305* origin and replication forks progressively moved outside the analyzed fragment, as indicated by the detection of bubble and large-Y intermediates. A similar pattern was observed for *dna2-1* mutants, although replication intermediate signals were fainter and moved away earlier, reflecting the faster S-phase onset in these cells observed by Fluorescence Activated Cell Sorting (FACS) analysis (data not shown). We failed to detect aberrant replication intermediates in *dna2-1*, or *dna2-1 exo1Δ* cells, suggesting that Dna2 function is dispensable to counteract gross fork abnormalities in checkpoint-proficient cells. However, Dna2 is likely to play a role in counteract fork defects, reflected by the increased sensitivity to HU of *dna2-1* mutants.

We then analyzed the contribution of Dna2 to collapsed fork transitions by carrying out psoralen 2D gels in *rad53* and *rad53 dna2-1* cells bearing, or not, *exo1* deletion. We found that *rad53 dna2-1* cells accumulated X-shaped intermediates (Figure 4C, blue arrowheads), evidencing a role of Dna2 in promoting putative reversed fork processing. However, the proportion of unresected cruciforms was lower in *rad53 dna2-1* cells when compared to *rad53 exo1* mutants (Figure 4D), suggesting that Dna2 is less efficient than Exo1 in promoting reversed fork resection. 2D gel profiles and X-spike/cone signal ratios were comparable in *rad53 exo1Δ dna2-1* and *rad53 exo1Δ* cells, suggesting that Dna2 does not extensively contribute to reversed fork resection when Exo1 is absent. Due to this epistatic behavior, we envisage that Dna2 may facilitate Exo1 access to lagging strand ends by removing Okazaki fragment 5′ flaps (Figure 4E).

### Sae2 and Exo1 interplay in collapsed fork metabolism and in counteracting chromosome instability

**Figure 5.**
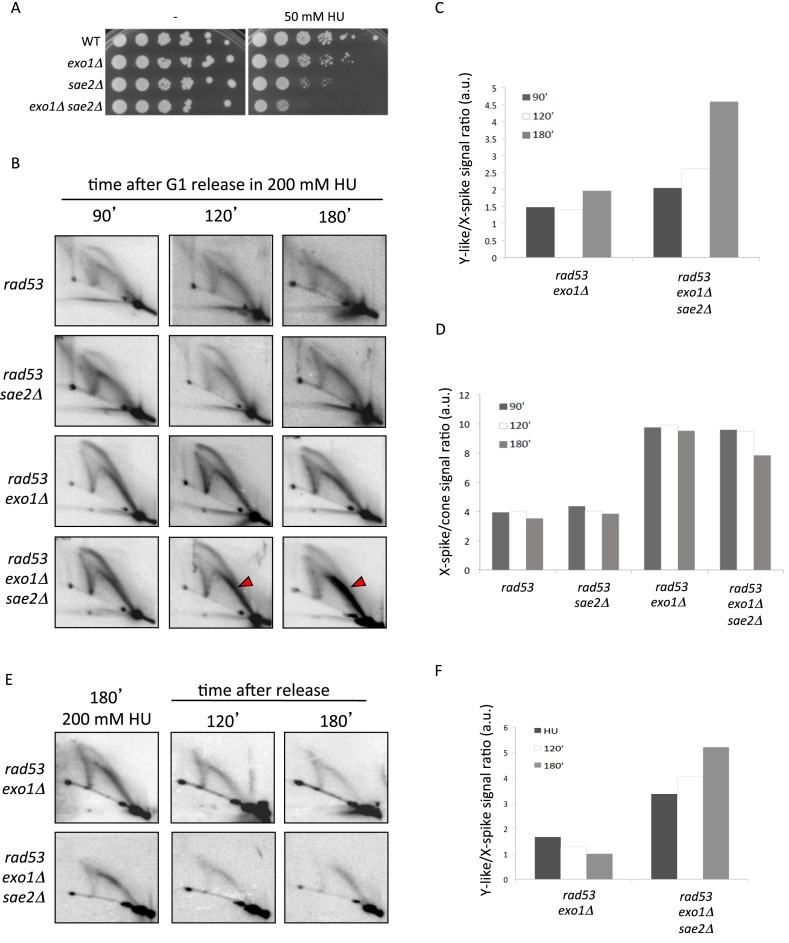
Sae2 counteracts aberrant transitions at Exo1-resected collapsed forks. (**A**) Serial dilutions of WT*, exo1Δ, sae2Δ* and *exo1Δ sae2Δ* cells plated on YPD in the absence (-) or presence of 50 mM HU. (**B**) 2D gel analysis of *rad53, rad53 sae2Δ, rad53 exo1Δ* and *rad53 exo1Δ sae2Δ* cells at the indicated times after G1 release into S-phase in the presence of 200 mM HU. Arrowheads indicate Y-like signals in *rad53 exo1Δ sae2Δ* cells. (**C**) Histogram plot of the ratios of Y-like and X-spike signals quantified from 2D gels shown in panel B. (**D**) Histogram plot of the ratios of X-spike and cone signals quantified from 2D gels shown in panel B. (**E**) 2D gel analysis of *rad53 exo1Δ* and *rad53 exo1Δ sae2Δ* cells at the indicated times after release from a HU-induced replication block. (**F**) Histogram plot of the ratios of Y-like and X-spike signals quantified from 2D gels shown in panel E.

Our genetic analysis revealed the synergistic HU sensitivity conferred by *exo1Δ* and *sae2Δ* alleles (Figure 5A), suggestive of interplay between these nucleases in promoting stalled fork functionality. Sae2 is a key nuclease in DSB repair that cooperates with the MRX (Mre11–Rad50–Xsr2) complex in DNA end resection (57,58). Sae2 and MRX are also required to stabilize replication forks approaching DSBs (30).

We analyzed the contribution of Sae2 to collapsed fork transitions in *rad53, rad53 sae2Δ, rad53 exo1Δ* and *rad53 exo1Δ sae2Δ* cells released into a synchronous S-phase in the presence of HU. We failed to observe relevant differences between the replication intermediates of *rad53* and *rad53 sae2Δ* cells (Figure 5B), suggesting that Sae2 does not contribute to collapsed fork processing when Exo1 is functional. Strikingly, *rad53 exo1Δ sae2Δ* cells accumulated high levels of Y-like intermediates when compared to *rad53 exo1Δ* mutants (red arrowheads). Y-like intermediates accumulation occurred seemingly at the expense of X-shaped molecules, the levels of which progressively decreased leading to Y-like structures being up to 4 times more abundant than reversed forks in *rad53 exo1Δ sae2Δ* cells (Figure 5C). These observations are consistent with Sae2 counteracting the accumulation of Y-like molecules as a result of unresected reversed fork processing. Noteworthy, X-spike to cone signal ratios of *rad53 exo1Δ* cells were not altered by *sae2* deletion (Figure 5D), suggesting that Sae2 is dispensable for fork processing when Exo1 is active.

We then analysed the impact of Sae2 ablation on the fate of collapsed forks following HU-block removal (Figure 5E). As described above, in *rad53 exo1Δ* cells unresected reversed forks and Y-like intermediates levels only slightly reduced following release from the replication block. In contrast, unresected reversed fork detection progressively decreased in *rad53 exo1Δ sae2Δ* cells, while Y-like shaped intermediates scarcely varied (Figure 5E). As a consequence, Y-like intermediates largely outnumbered intact X-shaped reversed fork molecules upon *sae2* deletion (Figure 5F). These findings suggest that Sae2 counteracts aberrant collapsed fork transitions by impeding putative reversed fork cleavage events. The nature of this Sae2 function at collapsed forks is intriguing. We note that *MRE11* deletion did not recapitulate the 2D gel phenotypes observed upon Sae2 ablation in *rad53 exo1Δ* (Supplementary Figure S2), suggesting that Sae2 acts at collapsed forks in a fashion different from its role on DSB repair and might process structures resembling its MRX-independent *in vitro* substrates (59).

**Figure 6.**
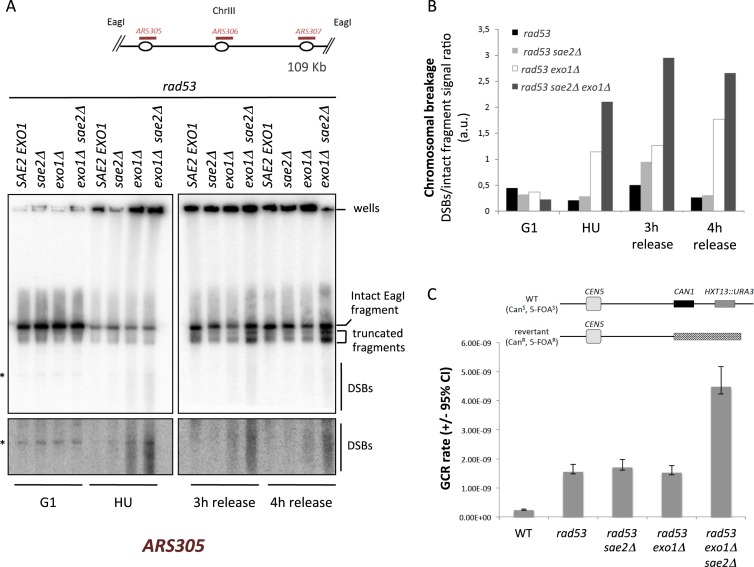
Exo1 and Sae2 cooperate in counteracting collapsed fork-related chromosomal breakage and spontaneous gross chromosomal rearrangements (GCR). (**A**) Pulsed field gel electrophoresis (PFGE) of EagI-digested chromosomes extracted from *rad53* (*SAE2 EXO1*), *rad53 sae2Δ* (*sae2Δ), rad53 exo1Δ* (*exo1Δ*) and *rad53 exo1Δ sae2Δ* (*exo1Δ sae2Δ*) cells arrested in G1 by α-factor treatment (G1), released into S-phase in 200 mM HU for 3 h (HU) or collected 3 and 4 h after release from the HU-block. Chromosomes were extracted in agarose plugs, digested with EagI, separated by PFGE and subjected to Southern blotting with an *ARS305*-specific probe. A higher exposure of the gel lower section containing DSB fragments is shown. An unspecific band is marked with an asterisk (*). The positions of wells, intact EagI fragments, truncated fragments and broken fragments (DSBs) signals are indicated. A schematic representation shows the relative positions on chromosome III of EagI restriction sites and early origins within the fragment. (**B**) Histogram plot of the ratios of DSBs and intact fragment signals quantified from the PGFE blots shown in panel A. (**C**) GCR rates in WT, *rad53, rad53 sae2Δ, rad53 exo1Δ* and *rad53 exo1Δ sae2Δ* cells. GCR rates were measured in exponentially growing cells bearing *CAN1* and *URA3* genes distal to *CEN5*.

We reasoned that cumulative cleavage events at collapsed forks upon impaired nucleolytic processing might have a profound impact on chromosome integrity. To test this hypothesis, we analyzed DSB formation along a 109 Kb EagI fragment containing forks emanating from the *ARS305, ARS306* and *ARS307* early and efficient replication origins by PFGE (60,61). *rad53, rad53 sae2Δ, rad53 exo1Δ* and *rad53 exo1Δ sae2Δ* cells were arrested in G1 and driven into a synchronous S-phase in the presence of HU for 3 h followed by release from the replication block in fresh medium. Irrespectively of their genotype, G1 arrested cells exhibited prominent unreplicated EagI fragments when analyzed with a probe for *ARS305* region (Figure 6A). Following HU treatment, the majority of *ARS305* signal localized to wells, reflecting chromosomal DNA stacking owing to the presence of replication intermediates and only a small fraction of intact linear fragments was detected. Of note, a smear of faster migrating DNA appeared in HU-treated *rad53 exo1* cells reflecting the formation of DSBs, which accumulated to a greater extent in *rad53 exo1 sae2* mutants (Figure 6A and B). A similar genetic dependency for chromosome fragmentation was observed when *ARS306* and *ARS307* regions or an EagI chromosomal fragment bearing the *ARS202* early origin were probed (Supplementary Figure S3A and B). Upon removal of the HU-induced replication block, detection of small chromosomal fragments in *rad53 exo1* and *rad53 exo1 sae2* persisted and two additional discrete bands corresponding to large molecules with molecular weight lower than the intact EagI fragment accumulated (Figure 6A). These truncated fragments, which might represent further DNA breakage or chromosomal rearrangements involving the analysed region, were most prominently detected in *rad53 exo1 sae2* cells.

These data suggest that Exo1 and Sae2 interface in suppressing DSBs occurring at stalled forks that prime chromosomal instability in checkpoint deficient cells. We thus tested whether Exo1 and Sae2 influenced GCR rates in *rad53* cells with an assay measuring the deletion of a region on chromosome V containing the *CAN1* gene and the *HTX13* locus replaced by *URA3* (14). We compared CGR rates of logarithmically growing WT, *rad53, rad53 exo1, rad53 sae2* and *rad53 exo1 sae2* cells (Figure 6C). In agreement with previous reports (14), WT cells exhibited a GCR rate of 2.30 × 10^−10^, which was increased 6.7-fold in *rad53-K227A* checkpoint deficient cells. Deletion of *exo1* or *sae2* genes did not significantly alter GCR rates in *rad53* cells. In contrast, double *exo1 sae2* deletion lead to a further 3-fold increase in GCR rates, indicating that Exo1 and Sae2 cooperate in suppressing chromosomal rearrangements in checkpoint deficient cells. Taken together with our previous findings, these data suggest that spontaneous fork collapse is more likely to give rise to GCRs in the absence of Exo1 and Sae2.

**Figure 7.**
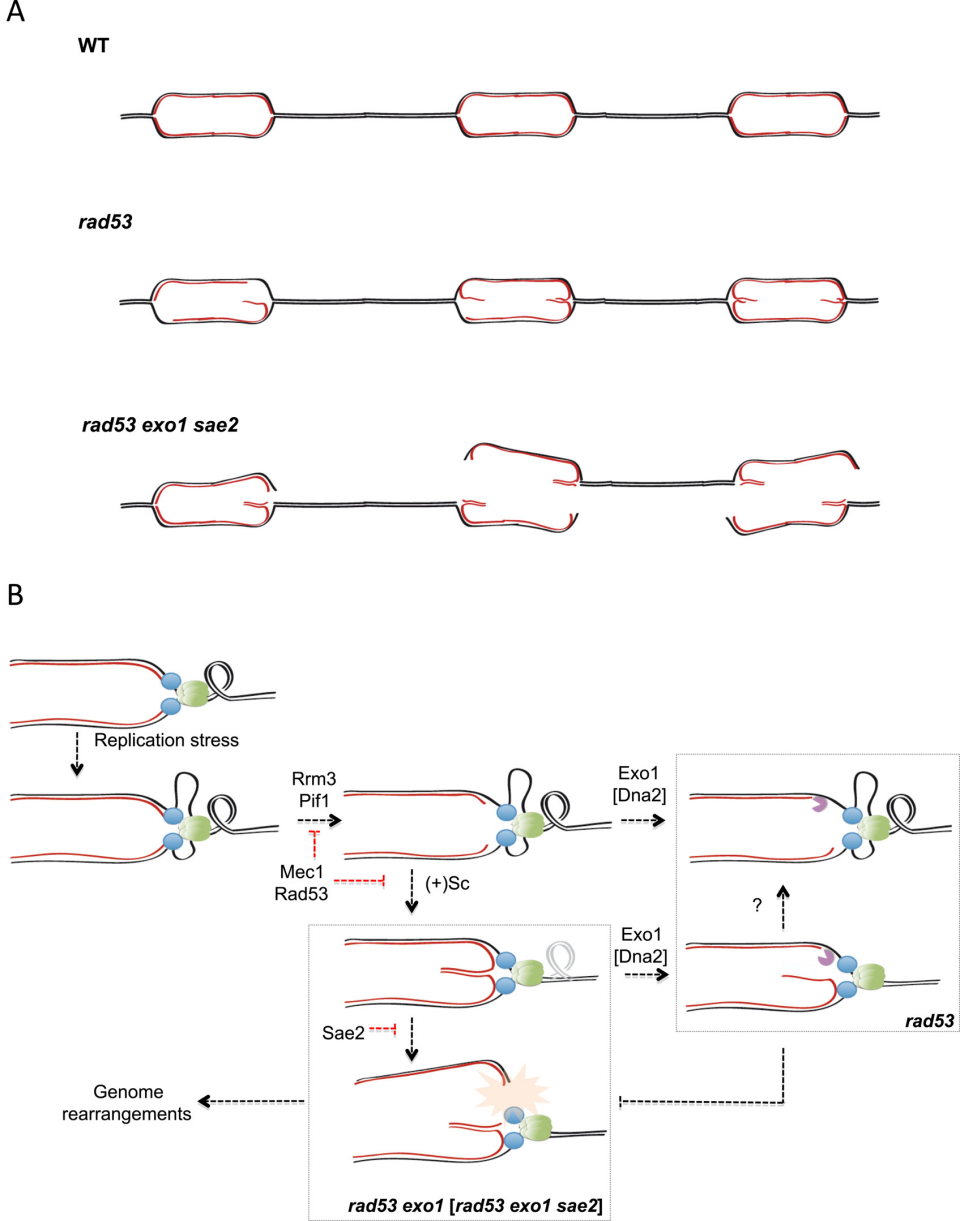
Collapsed fork transitions and chromosome fragility in checkpoint deficient cells. (**A**) A model for the contribution of collapsed fork transitions to the suppression of chromosome instability in cells experiencing replication stress. Nascent strand resection in checkpoint deficient cells (*rad53*) removes four-way branched reversed forks thus avoiding cleavage events generating Double Strand DNA Breaks (DSBs). Fork cleavage events in the absence of Exo1 and Sae2 function (*rad53 exo1 sae2*) can occur at adjacent replicons and lead to excision of chromosomal fragments and generation of branched chromosomal extremities potentially priming complex chromosomal rearrangements. (**B**) Nucleolytic processing by Exo1/Dna2 and Sae2 influences the fate of collapsed replication forks. Replication defects determine fork stalling and uncoupling between DNA unwinding by replicative helicases and DNA synthesis at leading and lagging strands. Mec1 and Rad53 protect stalled fork/replisome integrity thus avoiding the abnormal exposure of nascent strand ends and the accommodation of positive supercoiling by nascent strand annealing to conform reversed fork structures. In checkpoint deficient cells Exo1 resects lagging strands 5′ ends thus clearing reversed forks by converting them into structures resembling canonical forks and bearing extended ssDNA stretches. In the absence of Exo1, unresected reverse forks give raise to Y-like shaped molecules, likely though branch cleavage reactions counteracted by Sae2. Branch cleavage of reversed forks introduces physical discontinuities in replicating chromosomes that prime genome rearrangements.

The genetic dependency on Exo1 and Sae2 function of DSB formation upon HU treatment and increased GRC rates mirrors that of Y-like shaped molecules accumulation at collapsed forks. This suggests that in checkpoint deficient cells fork cleavage events might be related to chromosomal breaks formation and that Exo1 and Sae2 mediated processing events counteract chromosomal instability originating at collapsed forks (Figure 7A). Hence, we propose that Exo1 and Sae2 cooperate to eliminate collapsed fork structures (e.g. unresected reversed forks) otherwise susceptible of nucleolytic cleavage and in this way counteract chromosomal fragmentation and gross rearrangements. Furthermore, our data indicate that a network of nucleases co-ordinately processes collapsed replication forks to eliminate aberrant structures potentially priming genomic instability onset.

## DISCUSSION

The data here presented establish a direct link between stalled fork nucleolytic processing, DSB formation and chromosomal rearrangements. We found that, in checkpoint deficient cells, concerted fork processing prevents the accumulation of aberrant intermediates, some of which likely prime DSBs and chromosome instability. Exo1, with the assistance of Dna2, primarily processes reversed lagging strands. Exo1/Dna2-mediated resection counteracts the accumulation of aberrant Y-like molecules, which we propose represent reversed fork cleavage products. Sae2 cooperates with Exo1 in preventing the accumulation of these aberrant intermediates and thus avoid fork-related DNA breaks, which might influence the occurrence of chromosomal rearrangements in checkpoint deficient cells.

In cells experiencing replication stress, checkpoint kinases counteract extensive uncoupling between DNA synthesis and DNA unwinding by replicative helicases and the accumulation of torsional stress ahead of replication forks (19,62) (Figure 7B). These changes in fork architecture and topology likely promote replication intermediates transitions such as the annealing of nascent strands (i.e. fork reversal) and might abnormally expose DNA ends for exonucleolytic processing. Exposure of nascent strand ends is also likely favored by the unrestrained action of the Rrm3 and Pif1 helicases, inhibited upon checkpoint kinase activation (18). Exo1 is a key factor influencing the fate of collapsed replication forks, which bears a 5′ to 3′ exonuclease activity (63) and is hence thought to resect 5′ ends abnormally exposed at stalled lagging strands. The evidence here presented suggests that nascent strands engaged in reversed forks are a key substrate for Exo1 and that reversed fork processing might be an important step for preventing deleterious fork transitions.

Our data indicate that Dna2 promotes Exo1-mediated fork processing. In analogy with the role played by Dna2 in Okazaki fragment maturation (51,52), it is reasonable to envisage that Dna2 removes long flaps accumulating upon Okazaki fragment displacement and thus facilitate Exo1 access to lagging strand 5′ ends (Figure 4E). Such a function is consistent with the observed epistatic defects of Exo1 and Dna2 ablation on reversed fork resection. Dna2 has also been shown to counteract the accumulation of reversed forks in *Schizosaccharomyces pombe* and human cells exposed to HU-induced replication stress (64,65). The fission yeast Rad53 homolog Cds1 phosphorylates Dna2 likely enhancing its nuclease activity. Upon checkpoint-dependent activation, Dna2 was proposed to limit fork reversal by forming gaps precluding homology-driven base pairing of the nascent strands (64,66). Though we cannot exclude a similar role in counteracting fork reversal in checkpoint proficient cells, our data indicating that nascent strand resection may take place following fork reversal suggest that budding yeast Dna2 primarily limits reversed fork abundance by favoring their resection by Exo1.

In the absence of Exo1-mediated resection, checkpoint deficient cells accumulate Y-like shaped intermediates. Although other possibilities cannot be excluded, we propose that these are the product of reversed fork branch cleavage events (Figure 7B). This hypothesis is supported by the observations that (i) Y-spike molecules shape is consistent with the cleavage of new synthesized/parental branches at reversed forks, (ii) genetic contexts in which Y-spike intermediate levels increase also evidence a reduction of X-spike molecules and (iii) accumulation of Y-spike intermediates putative cleavage products correlates with breaks at regions containing stalled replication forks in *exo1* and *exo1 sae2* ablated checkpoint deficient cells. Different nucleases have been implicated in cleaving four-way branched structures and replication forks to render similarly shaped molecules (46,49,67–69). For instance, it has been proposed that Mus81 cleaves reversed forks both in checkpoint defective fission yeast cells (68) and in oncogene-overexpressing human cells (70). Our analysis failed to reveal a dependency of Y-like structure accumulation on Mus81. It is possible that other nucleases act redundantly with Mus81 in this context, thus masking the contribution of *mus81* deletion to reversed fork cleavage. Of note, combined ablation of Mus81, Yen1 and Slx1 SSE nucleases did not impact on Y-like molecule accumulation, suggesting that additional nucleases may be implicated. Further work will be required to characterize the activities and molecular mechanism mediating Y-like intermediates formation.

We found that the putative transitions leading to Y-spike molecule formation are favored upon *sae2* deletion. A straightforward interpretation for this observation is that Sae2 may act on collapsed forks in a fashion that counteracts branch cleavage events. The molecular function exerted by Sae2 in this context is enigmatic. The fact that *mre11* deletion does not recapitulate this effect suggests that Sae2 might act in a different fashion to the one exerted during DSB end processing. It has been recently shown that Sae2 can act on certain structures *in vitro* independently of Mre11, for instance by introducing nicks DNA hairpins (59) and that hairpin loops form at RPA-coated ssDNA (71). It is tantalizing to speculate that similar hairpin-like structures may form at RPA-coated ssDNA stretches within reversed forks and that their processing by Sae2 might somewhat induce changes in conformation precluding reversed fork cleavage. Cumulative branch cleavage events within a replicon would eventually fragment replicating chromosomes (Figure 7A). Noteworthy, the complex arrangement of DSBs so generated at collapse forks would bear a high potential for priming genomic rearrangements. In consistence with this notion, our data indicate that Y-like molecule and DSB accumulation in checkpoint compromised cells upon fork collapse both genetically depend on Exo1 and Sae2 function and correlate with increased GCR rates in unperturbed cells.

Our data also reveal a dual role for replication fork nucleolytic processing. Genetic evidence points at a redundancy between Exo1 and Mus81 in promoting viability upon HU-induced fork stalling, which would be independent of the role of Mus81 shared with Yen1. Likewise, Exo1 interplays with Dna2 and Sae2 in promoting the viability of checkpoint proficient cells experiencing replication stress. This evidence suggests that cooperative nucleolytic editing is required to promote stalled fork stability and/or late repair. This may reflect a necessity to trim replication intermediates and avoid their engagement in aberrant transitions precluding the completion of chromosome replication. Of note, impairment of Exo1, Mus81, Dna2 or Sae2 nucleases did not lead to evident replication intermediate defects in checkpoint proficient cells as observed by 2D gels, suggesting that their essential functions at stalled forks do not involve major fork transitions. Conversely, nucleolytic processing of collapsed forks does not seem to promote fork functionality, as ablation of these nucleases in checkpoint deficient cells does alter viability upon HU treatment (Supplementary Figure S4). Hence, in checkpoint deficient cells, nucleolytic processing is likely important to eliminate aberrantly shaped intermediates and thus limit their deleterious impact on chromosome integrity. A possible explanation for the apparently opposed roles exerted by nucleases upon fork stalling or upon fork collapse may lay in their regulation. Fork-relevant nucleases (i.e. Exo1, Mus81, Dna2 and Sae2/CtIP) are phosphorylated and regulated by cell cycle and checkpoint kinases both in yeast and mammalian cells (42,64,72–75). It is hence likely that, upon checkpoint defects, nucleases shift from exerting a fork protective effect to mediating the clearance of abnormal intermediates potentially priming rearrangements. This processing might not be specifically regulated by checkpoint kinases, but be instead generally relevant in conditions associated with reversed fork accumulation. Fork reversal has been observed in checkpoint-proficient human cells subjected to replication stress (76) (and references therein), but likely requires checkpoint inactivation or topology locking upstream of replication forks in yeast cells (19,77). This difference may reflect the necessity for yeast-specific checkpoint requirement to alleviate topological problems at stalled forks and/or that the formation of intermediates upstream of reversal (i.e. stable accumulation of ssDNA at forks) might be favored in yeast only upon fork collapse and thus limit the efficiency of re-priming following fork stalling (10,78).

Stalled fork nucleolytic processing might be particularly relevant for genomic instability onset in precancerous cells, which are characterized by oncogene-induced replicative stress. Importantly, reversed forks are prevalent intermediates in cells undergoing oncogenic stress (70), in which they have been proposed to act as ‘safe structures’ protecting the replication machinery in replication stress conditions (76) and nucleolytic cleavage of reversed forks was recently reported in this context (70). Hence, replication fork reversal and processing might be a key event during the malignant transformation of precancerous lesions, which might occur concomitantly to checkpoint-attenuating mutations. The mechanisms governing collapsed fork fates in pre-malignant cells might therefore deserve detailed investigation.

## Supplementary Material

SUPPLEMENTARY DATA
