# Supplementary material for: Nucleolytic processing of aberrant replication intermediates by an Exo1-Dna2-Sae2 axis counteracts fork collapse-driven chromosome instability
Source: Nucleic Acids Res. 2016 Sep 26;44(22):10676–90. doi: 10.1093/nar/gkw858 (PMC5159547; doi:10.1093/nar/gkw858)
Supplement: SUPPLEMENTARY DATA [file supp_44_22_10676__index.html]

Nucleolytic processing of aberrant replication intermediates by an Exo1-Dna2-Sae2 axis counteracts fork collapse-driven chromosome instability — Nucleolytic processing of aberrant replication intermediates by an Exo1-Dna2-Sae2 axis counteracts fork collapse-driven chromosome instability — SUPPLEMENTARY DATA 

# Nucleolytic processing of aberrant replication intermediates by an Exo1-Dna2-Sae2 axis counteracts fork collapse-driven chromosome instability

## SUPPLEMENTARY DATA

- SUPPLEMENTARY DATA
