# Supplementary material for: Nucleolytic processing of aberrant replication intermediates by an Exo1-Dna2-Sae2 axis counteracts fork collapse-driven chromosome instability
Source: Nucleic Acids Res. 2016 Sep 26;44(22):10676–90. doi: 10.1093/nar/gkw858 (PMC5159547; doi:10.1093/nar/gkw858)
Supplement: SUPPLEMENTARY DATA [file supp_gkw858_nar-02412-x-2016-File003.pdf]

Supp figure 1

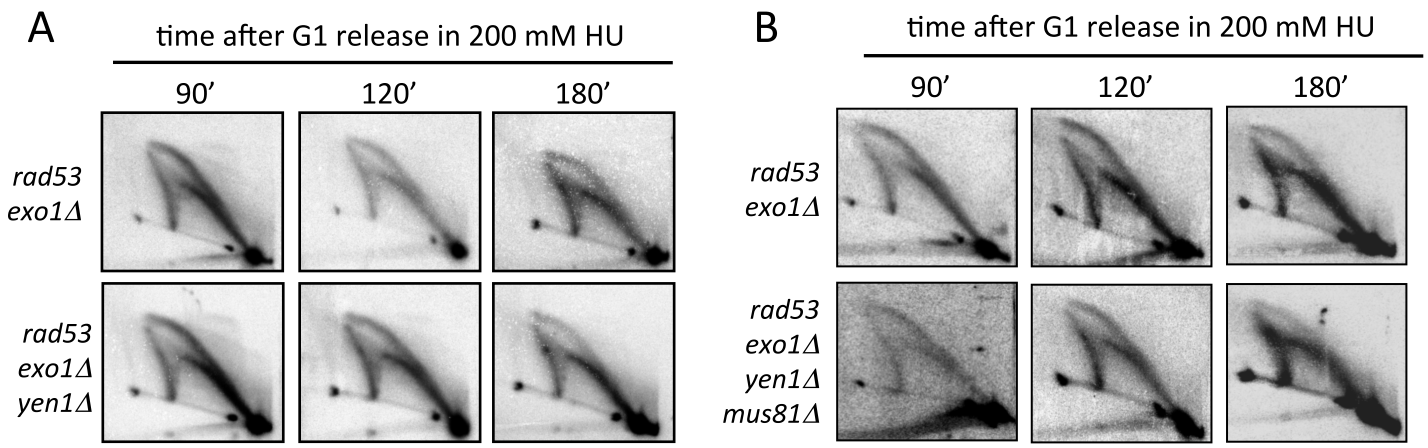

Supp. Figure 2

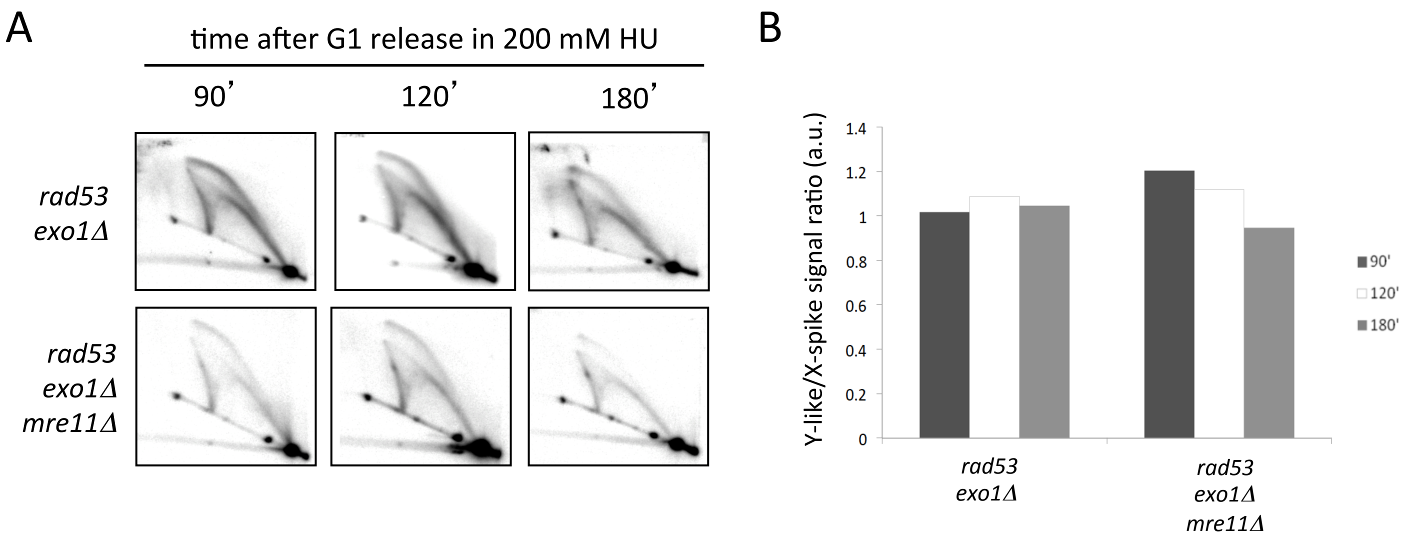

Supplementary Figure 3

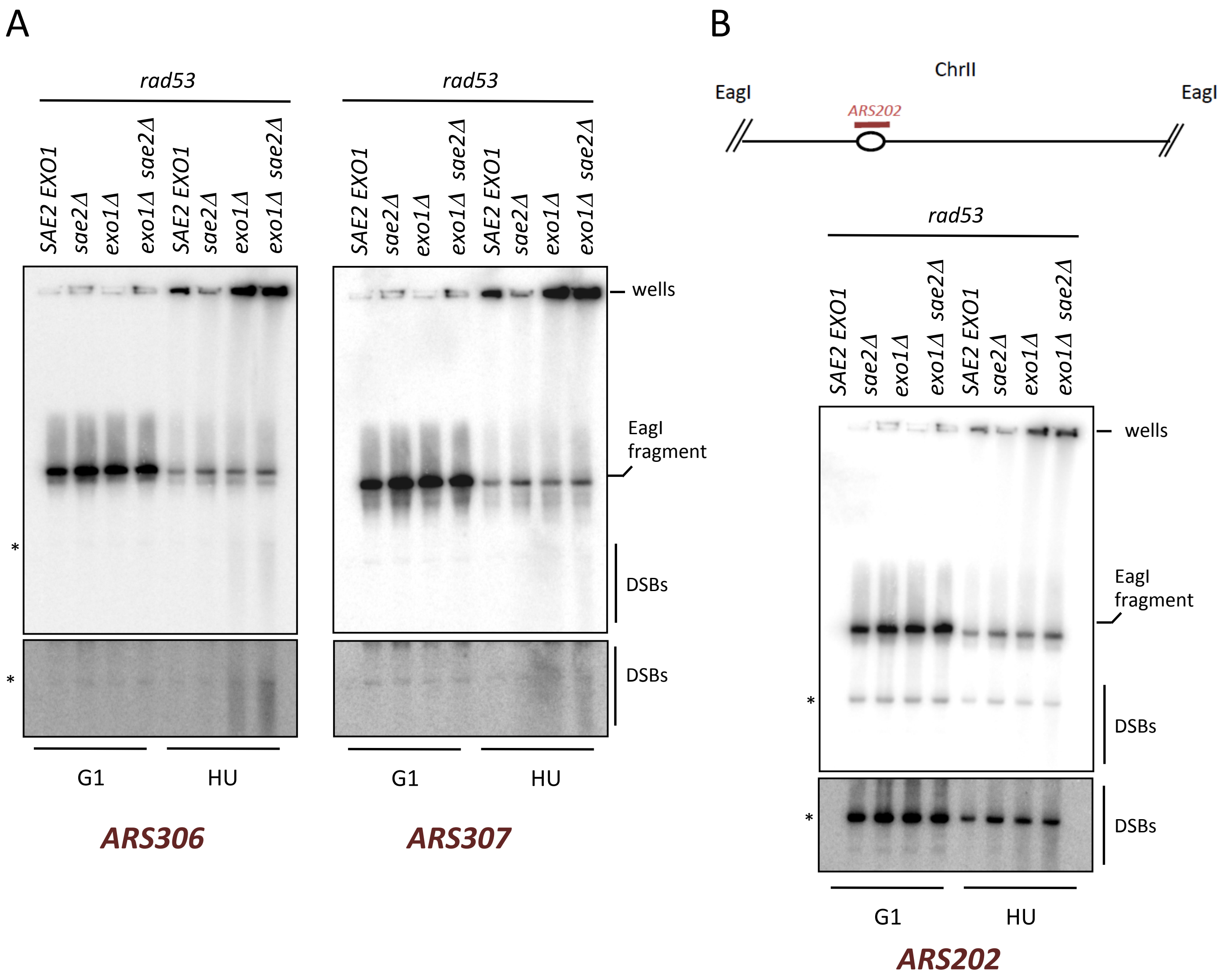

Supplementary Figure 4

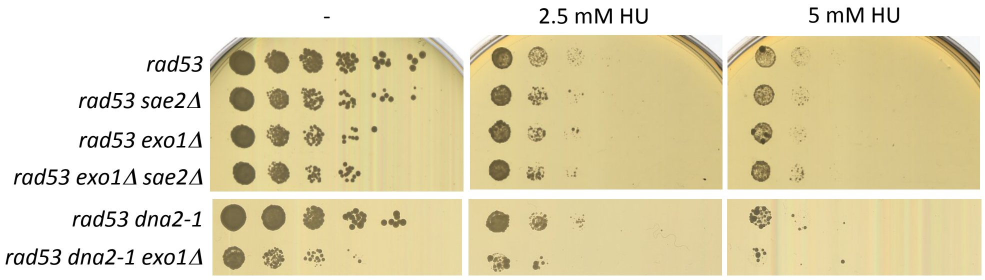

Supplementary Figure 1. Mus81 and Yen1 are dispensable for collapsed fork transitions. (A) 2D gel analysis of replication intermediates in *rad53 exo1Δ* and *rad53 exo1Δ yen1Δ* cells at the indicated times after G1 release into S-phase in the presence of 200 mM HU. (B) 2D gel analysis of replication intermediates in *rad53 exo1Δ* and *rad53 exo1Δ mus81Δ yen1Δ* cells at the indicated times after G1 release into S-phase in the presence of 200 mM HU.

Supplementary Figure 2. Mre11 does not contribute to collapsed fork processing. (A) 2D gel analysis of replication intermediates accumulating in *rad53 exo1Δ* and *rad53 exo1Δ mre11Δ* cells at the indicated times after G1 release into S-phase in the presence of 200 mM HU. (B). Histogram plot of the ratios of X-spike and cone signals quantified from 2D gels shown in panel A.

Supplementary Figure 3. Collapsed fork-related breakage at *ARS306*, *ARS307* and *ARS202* chromosomal regions. PFGE analysis of EagI-digested chromosomes extracted from *rad53* (*SAE2 EXO1*), *rad53 sae2* (*sae2Δ)*, *rad53 exo1Δ* (*exo1Δ) and rad53 exo1Δ sae2Δ* (*exo1Δ sae2Δ*) cells arrested in G1 by a-factor treatment (G1), released into S phase in 200 mM HU for 3 hours (HU), or collected 3 and 4 (4h release) hours after release from the HU-block. Chromosomes were extracted in agarose plugs, digested with EagI, separated by PFGE and subjected to Southern blotting with probes recognizing *ARS306* (A, left panel), *ARS307* (A, right panel) or ARS202 (B). The position of the wells, the intact chromosomes, the intact digested fragment and broken fragments (DSBs) signals are indicated. A schematic representation of the relative positions on chromosome II of EagI restriction sites and *ARS202* is shown.

Supplementary Figure 4. Sensitivity to low HU concentrations of checkpoint deficient cells bearing nuclease mutations. Serial dilutions of *rad53-K227A* (*rad53*), *rad53-K227A sae2Δ*, *rad53-K227A exo1Δ, rad53-K227A exo1Δ sae2Δ*, *rad53-K227A dna2-1* and *rad53-K227A dna2-1 exo1Δ* cells plated on YPD in the absence (-) or presence of 2.5 and 5 mM HU

Supplementary Table 1. Yeast strains used in this study.

Strain	Number	Genotype	Source
WT	CY7028	*MATa ADE2+ CAN1+, ura3-1, his3-11, leu2-3,112, trp1-1, RAD5+, DUN1::DUN1-3HA-TRP1*	Lab collection
rad53	CY7031	*MATa ADE2+ CAN1+, ura3-1, his3-11, leu2-3,112, trp1-1, RAD5+, DUN1::DUN1-3HA-TRP1, rad53K227A Kan-r*	Lab collection
exo1Δ	CY10342	*MATa ADE2+ CAN1+, ura3-1, his3-11, leu2-3,112, trp1-1, RAD5+, DUN1::DUN1-3HA-TRP1, exo1::HIS*	This study
rad53 exo1Δ	CY10343	*MATa ADE2+ CAN1+, ura3-1, his3-11, leu2-3,112, trp1-1, RAD5+, DUN1::DUN1-3HA-TRP1, rad53K227A Kan-r, exo1::HIS*	This study
rad53 GAL1::HA-EXO1	RB2230	*MATa, ade2-1, ura3-1, his3-11, leu2-3, 112 trp1-1, can1-100, RAD5+, DUN1::DUN1-3HA-TRP1, EXO1::His3MX6-GAL1-HA-EXO1, rad53K227A Kan-r*	This study
rad53 mus81Δ	CY10916	*MATa ADE2+ CAN1+, ura3-1, his3-11, leu2-3,112, trp1-1, RAD5+, DUN1::DUN1-3HA-TRP1, rad53K227A Kan-r, mus81::HPH*	This study
rad53 mus81Δ exo1Δ	CY10917	*MATa ADE2+ CAN1+, ura3-1, his3-11, leu2-3,112, trp1-1, RAD5+, DUN1::DUN1-3HA-TRP1, rad53K227A Kan-r, exo1::HIS, mus81::HPH*	This study
mus81Δ	CY10914	*MATa ADE2+ CAN1+, ura3-1, his3-11, leu2-3,112, trp1-1, RAD5+, DUN1::DUN1-3HA-TRP1, mus81::HPH*	This study
mus81Δ exo1Δ	CY10915	*MATa ADE2+ CAN1+, ura3-1, his3-11, leu2-3,112, trp1-1, RAD5+, DUN1::DUN1-3HA-TRP1, exo1::HIS, mus81::HPH*	This study
rad53 yen1Δ	CY10908	*MATa ADE2+ CAN1+, ura3-1, his3-11, leu2-3,112, trp1-1, RAD5+, DUN1::DUN1-3HA-TRP1, rad53K227A Kan-r, yen1::NAT*	This study
rad53 yen1Δ exo1Δ	CY10912	*MATa ADE2+ CAN1+, ura3-1, his3-11, leu2-3,112, trp1-1, RAD5+, DUN1::DUN1-3HA-TRP1, rad53K227A Kan-r, exo1::HIS, yen1::NAT*	This study
yen1Δ	CY10907	*MATa ADE2+ CAN1+, ura3-1, his3-11, leu2-3,112, trp1-1, RAD5+, DUN1::DUN1-3HA-TRP1, yen1::NAT*	This study

yen1Δ exo1Δ	CY10909	*MATa ADE2+ CAN1+, ura3-1, his3-11, leu2-3,112, trp1-1, RAD5+, DUN1::DUN1-3HA-TRP1, exo1::HIS, yen1::NAT*	This study
yen1Δ exo1Δ mus81Δ	CY10911	*MATa ADE2+ CAN1+, ura3-1, his3-11, leu2-3,112, trp1-1, RAD5+, DUN1::DUN1-3HA-TRP1, exo1::HIS, yen1::NAT, mus81::HPH*	This study
yen1Δ mus81Δ	CY10910	*MATa ADE2+ CAN1+, ura3-1, his3-11, leu2-3,112, trp1-1, RAD5+, DUN1::DUN1-3HA-TRP1, yen1::NAT, mus81::HPH*	This study
exo1Δ mus81Δ	CY10915	*MATa ADE2+ CAN1+, ura3-1, his3-11, leu2-3,112, trp1-1, RAD5+, DUN1::DUN1-3HA-TRP1, mus81::HPH, exo1::HIS*	This study
exo1Δ yen1Δ	CY10909	*MATa ADE2+ CAN1+, ura3-1, his3-11, leu2-3,112, trp1-1, RAD5+, DUN1::DUN1-3HA-TRP1, yen1::NAT, exo1::HIS*	This study
rad53 slx1Δ exo1Δ	CY12289	*MATa ADE2+ CAN1+, ura3-1, his3-11, leu2-3,112, trp1-1, RAD5+, DUN1::DUN1-3HA-TRP1, rad53K227A Kan-r, exo1::HIS, slx1::HIS*	This study
rad53 slx1Δ exo1Δ yen1Δ	CY12290	*MATa ADE2+ CAN1+, ura3-1, his3-11, leu2-3,112, trp1-1, RAD5+, DUN1::DUN1-3HA-TRP1, rad53K227A Kan-r, exo1::HIS, slx1::HIS, yen1::NAT*	This study
rad53 slx1Δ exo1Δ mus81Δ	CY12291	*MATa ADE2+ CAN1+, ura3-1, his3-11, leu2-3,112, trp1-1, RAD5+, DUN1::DUN1-3HA-TRP1, rad53K227A Kan-r, exo1::HIS, slx1::HIS, mus81::HPH*	This study
rad53 slx1Δ exo1Δ mus81Δ yen1Δ	CY12292	*MATa ADE2+ CAN1+, ura3-1, his3-11, leu2-3,112, trp1-1, RAD5+, DUN1::DUN1-3HA-TRP1, rad53K227A Kan-r, exo1::HIS, slx1::HIS, mus81::HPH, yen1::NAT*	This study
rad53 rad1Δ	CY11938	*MATa ADE2+ CAN1+, ura3-1, his3-11, leu2-3,112, trp1-1, RAD5+, DUN1::DUN1-3HA-TRP1, rad53K227A Kan-r, rad1::NAT*	This study
rad53 rad1Δ exo1Δ	CY11939	*MATa ADE2+ CAN1+, ura3-1, his3-11, leu2-3,112, trp1-1, RAD5+, DUN1::DUN1-3HA-TRP1, rad53K227A Kan-r, exo1::HIS, rad1::NAT*	This study
rad53 sae2Δ	CY11669	*MATa ADE2+ CAN1+, ura3-1, his3-11, leu2-3,112, trp1-1, RAD5+, DUN1::DUN1-3HA-TRP1, rad53K227A Kan-r, sae2::HPH*	This study
sae2Δ	CY11672	*MATa ADE2+ CAN1+, ura3-1, his3-11, leu2-3,112,*	This study

		trp1-1, RAD5+, DUN1::DUN1-3HA-TRP1, sae2::HPH	
sae2Δ exo1Δ	CY11671	*MATa ADE2+ CAN1+, ura3-1, his3-11, leu2-3,112, trp1-1, RAD5+, DUN1::DUN1-3HA-TRP1, exo1::HIS, sae2::HPH*	This study
rad53 exo1Δ sae2Δ	CY11670	*MATa ADE2+ CAN1+, ura3-1, his3-11, leu2-3,112, trp1-1, RAD5+, DUN1::DUN1-3HA-TRP1, rad53K227A Kan-r, exo1::HIS, sae2::HPH*	This study
rad53 mre11Δ	CY11877	*MATa ADE2+ CAN1+, ura3-1, his3-11, leu2-3,112, trp1-1, RAD5+, DUN1::DUN1-3HA-TRP1, rad53K227A Kan-r, mre11::NAT*	This study
mre11Δ	CY12254	*MATa ADE2+ CAN1+, ura3-1, his3-11, leu2-3,112, trp1-1, RAD5+, DUN1::DUN1-3HA-TRP1, mre11::NAT*	This study
mre11Δ exo1Δ	CY12256	*MATa ADE2+ CAN1+, ura3-1, his3-11, leu2-3,112, trp1-1, RAD5+, DUN1::DUN1-3HA-TRP1, mre11::NAT, exo1::HIS*	This study
rad53 mre11Δ exo1Δ	CY11878	*MATa ADE2+ CAN1+, ura3-1, his3-11, leu2-3,112, trp1-1, RAD5+, DUN1::DUN1-3HA-TRP1, rad53K227A Kan-r, exo1::HIS, mre11::NAT*	This study
dna2-1	CY11836	*MATa ADE2+ CAN1+, ura3-1, his3-11, leu2-3,112, trp1-1, RAD5+, DUN1::DUN1-3HA-TRP1, dna2-1*	This study
exo1Δ dna2-1	CY11837	*MATa ADE2+ CAN1+, ura3-1, his3-11, leu2-3,112, trp1-1, RAD5+, DUN1::DUN1-3HA-TRP1, dna2-1, exo1::HIS*	This study
rad53 dna2-1	CY11788	*MATa ADE2+ CAN1+, ura3-1, his3-11, leu2-3,112, trp1-1, RAD5+, DUN1::DUN1-3HA-TRP1, rad53K227A Kan-r, dna2-1*	This study
rad53 exo1Δ dna2-1	CY11789	*MATa ADE2+ CAN1+, ura3-1, his3-11, leu2-3,112, trp1-1, RAD5+, DUN1::DUN1-3HA-TRP1, rad53K227A Kan-r, exo1::HIS, dna2-1*	This study
rad53 rad52Δ	CY 14133	*MATa ADE2+ CAN1+, ura3-1, his3-11, leu2-3,112, trp1-1, RAD5+, DUN1::DUN1-3HA-TRP1, rad53K227A Kan-r, rad52::HPH*	This study
hxt13::URA3	CY13804	*MATa ADE2+ CAN1+, ura3-1, his3-11, leu2-3,112, trp1-1, RAD5+, DUN1::DUN1-3HA-TRP1, hxt13::URA*	This study
hxt13::URA3 rad53	CY 13811	*MATa ADE2+ CAN1+, ura3-1, his3-11, leu2-3,112, trp1-1, RAD5+, DUN1::DUN1-3HA-TRP1, rad53K227A*	This study

		Kan-r, hxt13::URA, rad53K227A Kan-r,	
hxt13::URA3 rad53 exo1Δ	CY 13813	*MATa ADE2+ CAN1+, ura3-1, his3-11, leu2-3,112, trp1-1, RAD5+, DUN1::DUN1-3HA-TRP1, rad53K227A Kan-r, hxt13::URA, rad53K227A Kan-r,exo1::HIS*	This study
hxt13::URA3 rad53 sae2Δ	CY13815	*MATa ADE2+ CAN1+, ura3-1, his3-11, leu2-3,112, trp1-1, RAD5+, DUN1::DUN1-3HA-TRP1, rad53K227A Kan-r, hxt13::URA, rad53K227A Kan-r, sae2::HPH*	This study
hxt13::URA3 rad53 sae2Δ exo1Δ	CY 13817	*MATa ADE2+ CAN1+, ura3-1, his3-11, leu2-3,112, trp1-1, RAD5+, DUN1::DUN1-3HA-TRP1, rad53K227A Kan-r, hxt13::URA, rad53K227A Kan-r, sae2::HPH, exo1::HIS*	This study
